# Effectiveness of a mixed lifestyle program in couples undergoing assisted reproductive technology: a study protocol

**DOI:** 10.1186/s12978-023-01652-6

**Published:** 2023-08-01

**Authors:** Padideh Malekpour, Robab hasanzadeh, Mojgan Javedani Masroor, Reza Chaman, Zahra Motaghi

**Affiliations:** 1 Shahroud University of Medical Sciences, Shahroud, Iran; 2 Department of Midwifery, Bonab Branch, Islamic Azad University, Bonab, Iran; 3 School of Medicine, Shahid Akbar-Abadi Clinical Research Development Unit (Sh A C R D U), Iran University of Medical Sciences, Tehran, Iran; 4 Department of Epidemiology, School of Health, Shiraz University of Medical Sciences, Shiraz, Iran; 5 Department of Reproductive Health, School of Nursing and Midwifery, Shahroud University of Medical Sciences, Shahroud, Iran

**Keywords:** Infertility, Health-promoting lifestyle, Assisted reproductive technology

## Abstract

**Background:**

The desire for fertility is the manifestation of yearning for immortality. Infertility and assisted reproductive technology (ART) expose couples to great affective, anxiety, stress, and financial burden. Increasing evidence emphasize the impact of lifestyle on infertility. One of the most crucial factors affecting the fertility process is the nutrition patterns, the amount and quality of physical activities, emotional problems management; modulate stressors, relief from anxiety, and the living conditions of couples. Most ART treatment interventions in Iran are not integrated into lifestyle programs. Therefore, this research will investigate the impact of mixed fertility health-promoting programs in couples who use ARTs.

**Methodology/Design:**

This study entails three steps. The first step includes the systematic review of literature on a health-promoting lifestyle in infertile couples undergoing ARTs, a systematic review of observational studies and interventions in couple’s lifestyle, then, a systematic review of qualitative studies on infertility in couples and their lifestyle, and in the final step couple’s life style literature systematically will evaluate in Iran. In case of failure to obtain the required results from systematic reviews, cross-sectional studies will be carried out to determine the lifestyle of infertile couples receiving ARTs. In the second stage, by holding a panel of experts, an intervention is planned based on the results of the previous stages in order to improve the lifestyle of couples. In the final step, the designed intervention will be administered as a random clinical trial—on ART candidates, in intervention or control groups in one of Iran University of Medical Sciences hospitals. Afterward, the data’s will be evaluated by using standard questionnaires, that include health-promoting lifestyle questionnaire (HPLII), Beck’s depression inventory (BDI), international physical activity questionnaire—short form (IPAQ-SF), and food frequency questionnaire (FFQ). The statistical analysis will be carried out in SPSS software. During the study, subjects meeting the inclusion criteria were randomly selected and randomized into the intervention and control groups. The health-promoting lifestyle training program will be executed for the intervention group while the standard care program is administered to the control group. The content of this program will be obtained from the consensus opinions of the expert panel. The program includes diet recommendations, physical activity, and stress management. Appropriate time, frequency, duration and number of activities will be considered. Communication with subjects will be possible through private meeting special comfort room. Support to the participants will also be through clinical visits social media, SMS and phone calls. Nutritional changes, physical activity amount, anxiety and stress level, abdominal circumference (AC), and body mass index (BMI) will be measured after the completion of the specified time interval. The initial outcome includes examining chemical pregnancy (2 weeks after the transmission) and clinical pregnancy by ultrasound (6 weeks after). The secondary outcome will be live birth rate. Retrieved oocyte and embryo numbers will also be reported.

**Discussion:**

Health-promoting lifestyle programs are essential in assisted reproductive technologies to improve pregnancy results and live birth. These programs in association with in-vitro fertilization (IVF) influence the outcome of fertilization. In addition, enhancing parental health leads to healthy pregnancy outcome. Despite the frequency of lifestyle risk factors, employing proper methods helps reduce anxiety and stress, modify dietary patterns, and perform qualitatively and quantitatively balanced physical activities. In addition, having coping skills and mental health management methods, in nowadays modern world challenges seems crucial and effective in solving fertility problems and reducing them before pregnancy.

## Plain Language Summary

For most people, children are the meaning of life. In some cultures, children are regarded as a family function and thus infertility is displeasing problem. In public believes, children are divine blessings. Not having children signifies a sick body and incomplete identity, which give rise to psychological and socio-economic consequences. Studies show that fertility and childbearing are biomarkers of survival and affect infertile men and women subconsciously. Since childbearing is one of the most crucial pillars of human marital life, an increase in infertile cases and use of ARTs by infertile couples, the lack of physical activity, sedentary lifestyle, unhealthy diet, stress, anxiety, and psychological problems caused by infertility problems, and the side effects of using ARTs preoccupy couples and distract their attention from healthy lifestyle and well-being issues. Despite the crucial importance of prevention in the modern world and the cost-effectiveness of prevention till treatment, numerous research findings expressing the importance of health education and disease ameliorates. These dimensions are still overlooked. Considering the importance of the issue, to the reduction of damages caused by chronic diseases, such as preventable NCDs problems including infertility, to promote fertility results, improving before conception. Oocyte retrieving time and in window period could improve embryo-maternal outcomes. Ensures couples’ health, increases individuals’ life satisfaction, and promotes health and lifespan of individuals, which is the ultimate goal of the healthcare system are the other purpose of health promoting process. It seems that health promotion lifestyle programs in Iran for infertile couples have not yet been integrated. Therefore, the combination of these programs with ART can be effective in achieving the goals of improving reproductive health. This research seeks to promote programs based on improving the health of infertile couples, increasing childbearing and increasing live births in people who are candidates for ART. A mixed study in the first step will be carried out by a systematic review of studies on ART candidate couples ‘lifestyles. If the desired results are not achieved, a cross-sectional study will be conducted. In the second step, an intervention will be designed and developed to execute the lifestyle program following the specialists’ opinions concerning nutrition, physical activity, and stress management. The next step will be carried out by implementing RCT. This intervention will be executed and evaluated at the end.

## Background

Amongst all steps in the lifecycle of a living creature, the highest evolutionary pressure is exerted on the process of fertility [1]. The most recent reports indicate a decrease in the live birth rate [2]. Considering the rising rate of infertility [3] and the need to use ART, lifestyle factors are more emphasized. The WHO defined infertility as a disease in the male or female reproductive system, which failed to achieve pregnancy after 12 months of unprotected sexual intercourse. In total, 186 million people and 48 million couples are infertile worldwide [4]. Infertility has destructive social and health consequences including social stigma, economic problems, and poor physical and mental well-being [5]. Following a meta-analysis report, the lifetime prevalence of infertility amounted to (3.3–21.3%) in the Eastern Mediterranean Region, 7% in England, and 30% in African countries [4, 6]. The population problem is a fundamental issue concerning policy-making and future planning. Based on the 2019–2020 censuses, the total fertility rate in Iran decreased to 1.2 children per woman. Taking into account the current fertility process, the population growth will approach zero between 2036 and 2041 [7]. ART comprises all methods in which oocytes, sperms, and embryos are directly manipulated outside the body in in-vitro. The first and most common form of ART is in vitro fertilization (IVF) [8]. The effects of lifestyle on infertility shown in the accumulated data from various studies suggest that such factors as smoking, depression, and anxiety can contribute to infertility [4]. Furthermore, mounting evidence indicated that obesity, an imbalanced diet, and a lack of physical activities play a crucial role in infertility causes [9]. Recent studies pointed out that inflammation in the body can cause damage to Germ cells. Following a diet rich in antioxidants, flavonoid resources, and unsaturated fatty acids, and decreasing the consumption of red meat, particularly processed meats, are effective in reducing inflammation. Sticking to a proper diet before pregnancy is an effective key solution [10]. Food-based dietary guidelines (FBDG) recommend reducing the sugar and saturated fat intake and increasing the use of unsaturated fat, seeds, whole grains, fruits, and fish [11]. The Mediterranean diet (MD) is one of the healthiest and most beneficial diets in the world. Food intake pattern in this diet includes large amounts of pulses, seeds, whole grains, fruits and vegetables, median intake of fish and wine, and low intake of dairy products and red meat. Fat intake resources are olive and olive oil. This diet is a rich source of unsaturated fats, fibers, antioxidants, and the minimum amount of saturated fats [12]. Vojokovic conducted a study in the Netherlands on the relationship between dietary styles and infertility and identified two dietary patterns. The first pattern (health conscious–low processed) emphasizes the high intake of fruits, vegetables, whole grains, and seeds and the low intake of processed foods, such as Mayonnaise, snacks, and meat products [13]. Noori’s study suggested that dairy consumption affects sperm parameters, whereas the consumption of saturated fats and calcium resulted in poor-quality sperm. On the other hand, the consumption of high amounts of folate and selenium, plus low consumption of cholesterol has a positive impact on the improvement of sperm parameters [14]. In their study, Xiao Cheng indicated that healthy diet adjuvant therapy, Mediterranean diet (MD), Ketogenic diet (KD), and dietary approaches to stop hypertension (DASH) were effective on fertility results [15]. Results of some studies demonstrated the impact of a sedentary lifestyle, and as a result, low health-related quality of life (HRQoL) on infertile people. Furthermore, high BMI and the increase in anxiety and stress affect HRQoL [16]. Even though exercise is regarded as a general improving factor, excessive exercise has an unpleasant and inhibitor impact on the hypothalamic–pituitary–adrenal axis (HTP axis). In the study by Minas, exercise reduced oxidative stress and improved chronic inflammation by increasing antioxidant defense in the testes. In addition, it decreased obesity, diabetes, and infertility rate [17]. The effective factors on the well-being and health of women with primary infertility were investigated in India and factors, such as psychological problems, duration of infertility, insufficient physical activity, education level, sleeping pattern, and family structure were shown to be crucially important in infertility [18]. The study by Palomba on 216 women under the treatment by IVF/ICSI revealed that regular physical activity before fertilization procedure improves fertility outcomes in infertile obese women [19]. Gaskin in the study of environment and fertility health (EARTH) examined the physical activity in 427 women under IVF treatment. The results showed that 2.8 h of average or severe physical activity per week has no significant impact on embryo implantation, clinical pregnancy, and live birth. However, women performing activities, such as aerobics, skiing, and boating had a higher rate of live birth [20]. In addition to the impact of exercise and physical activity on general health and fertility, it is quite clear that the type of food and diet styles is amongst the crucially important pillars of a healthy life, mental and psychological health, and psychological issues. Diagnosis of infertility is followed by comprehensive anxiety and stress and causes consequences, such as depression, unhappiness, guilty feeling, low self-esteem, destroyed self-confidence and inability to overcome obstacles and problems [21]. Research reported the anxiety rate caused by infertility amounts to approximately 75.9% in women and 60.6% in men. This high anxiety level and stress pressure affects couples’ fertility health [22]. Regardless of the impact of infertility on the stress and anxiety rate, IVF/ICSI procedures can cause stress and anxiety. Supporting couples in this step and providing them with the required stress and anxiety management training helps improve the quality of their life and increase the possibility of achieving their valuable wish’s goal [23]. Numerous factors affect lifestyle. Health-promoting lifestyle includes the following six dimensions: diet, exercise, stress management, health responsibility, interpersonal relationships, and spiritual growth leading to self-actualization. Lifestyle refers to all behaviors controlled by the individual, or behaviors that affect personal health risk factors [24]. A comprehensive approach suggests that health-protective behaviors (HPB) (risk reduction and prevention) and health-promoting behaviors can be regarded as two supplementary components of a healthy lifestyle. These behaviors, improve the health, self-actualization, and psychological maturity ‏of individuals. The European Society of Human Reproduction and Embryology (ESHRE) guideline argues that multipurpose programs in various fields are crucial to meet cognitive, communicative, and mental-psychological needs, as well as consultation concerning lifestyle [25]. Doomar conducted a study on 12,800 infertile women and found that IVF candidates have habits, such as smoking, drinking coffee and alcohol, having a sedentary lifestyle, and the use of recreational or unauthorized drugs [26]. Studies in Iran showed the prevalence of psychological disorders including depression, hypochondriasis, and paranoia among infertile couples [27], and these groups exhibited a high tendency toward unhealthy and Western nutrition [28]. Furthermore, the increase in exercise in women with IVF pregnancy was associated with a significant decrease in gestational diabetes and pre-eclampsia [29]. In the study by Baheiraei on the lifestyle of infertile couples, negligence towards a healthy lifestyle, particularly among men, indicates that this group needs appropriate health protection training programs before carrying out infertility treatments, as well as during and after IVF interventions [30]. A tailored literature-based program includes nutrition, physical activity, and exercise, together with stress reduction in the course of IVF/ICSI interventions. Similar integrated studies that include a combination of various health dimensions have rarely been conducted in Iran. The necessity of these programs in fertility clinics before beginning any medical treatment or procedure concerning the fertilization procedures is sensed and their absence can affect fertilization outcomes. It is hoped that this study can improve fertilization outcomes by promoting couples’ lifestyles.

## Aims of study

**Fig. 1:**
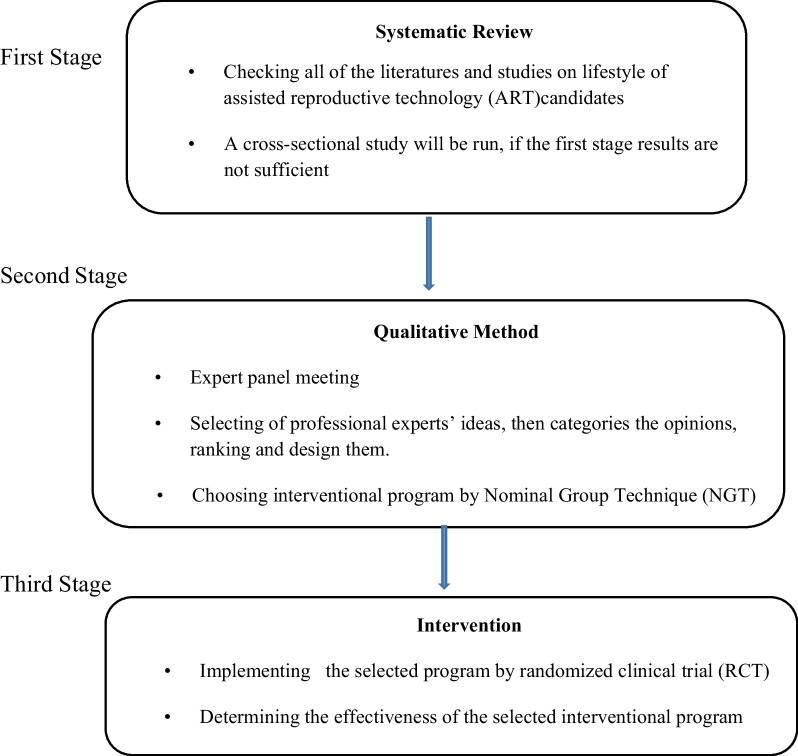
Study diagram

The main aim of the study in the first stage is to design a lifestyle intervention program in infertile couples receiving assisted reproductive technologies (ART). Then studies on the lifestyle of couples undergoing IVF/ICSI treatment will be identified and investigated. Choosing a suitable and beneficial program to promote health in men and women will be examined. After that, lifestyle risk factors (e.g., the lack of physical activity, unhealthy diet, consumption of fast foods and processed foods, weight gain and obesity, consumption of alcohol and coffee, smoking, use of other stimulants and narcotic drugs), psychological pressure complications (e.g., depression, stress, and anxiety), the lack of adequate alleviating training to manage stress and anxiety, sleep disorders, insomnia and sleep hygiene in couples will be investigated and studied. In the next step, an intervention will be executed and its effectiveness will be determined (Fig. 1).

## Specific objectives of study

### First stage

1. Identifying the lifestyle of infertile couples undergoing ARTs in the observational studies through a systematic review.
2. Identifying the lifestyle of infertile couples undergoing ARTs in the interventional studies through a systematic review.
3. Identifying the lifestyle of infertile couples undergoing ARTs in the qualitative studies through a systematic review.
4. Determining the impact of lifestyle-promoting interventions, concerning diet, on infertile couples undergoing ARTs using available studies.
5. Determining the impact of lifestyle-promoting interventions, concerning physical activity, on infertile couples undergoing ARTs using available studies.
6. Determining the impact of lifestyle-promoting interventions, concerning stress management, on infertile couples undergoing ARTs using available studies.

### Second stage (experts’ panel)

A survey will be conducted on expert’s advices and a lifestyle-promoting intervention for infertile couples undergoing ARTs will be designed by establishing an expert panel.

### Third stage

In this step, a mixed training program, attained based on the expert panel consensus in each selected health related dimensions, will be held in the form of a clinical trial to determine its effectiveness on retrieved oocytes number, sperm parameters, pregnancy rate, Live birth rate.

## Design and methodology

This research is conducted based on Standard Protocol Items (SPIRIT) [31].

The study entails three stages that start with a systematic literature review. A cross-sectional study will be carried out if the desired results are not achieved in the first stage. An expert panel will be held qualitatively to survey experts in various lifestyle fields. A consensus of opinions will be obtained after collecting the opinions of experts and professors in lifestyle dimensions. A tailored training program, developed based on the results of the previous step, will be designed. After selecting a suitable lifestyle training program for the couples undergoing ART, the impact of the intervention will be examined via clinical trial. The obtained data will be analyzed by SPSS software.

## Quantitative stage of study

In this stage, the eligible subjects who are interested to participate in the clinical trial will be randomized into intervention or control groups. The target group includes infertile couples undergoing ARTs. The age range of the participants is from 18 to 40 years. They must be able to read and write. It must be their first or second IVF or ICSI session.

## Sample size and sampling method

The sample size is estimate based on similar studies on the lifestyle of infertile couples [24]. The comparison formula between two ratios will be used at the 95% confidence level The Cronbach’s α of 0.05 and power of 80% will be considered. Finally, 600 couples will be included in the study, of which 150 female and 150 males will be placed in the lifestyle program group and the rest in the control group.

### Registering participants

Clients of the fertility clinics of Akbar Abadi Hospital in Iran University of Medical Sciences will be referred to the research unit after receiving an initial examination by the infertility clinic’s doctors and admitted by infertility midwives or nurses. The study will be described to the consenting participants. The demographic questionnaire and consent form will be completed by the participants and they will ensure of the confidentiality of their information.

### Inclusion criteria

Interest to participate in the research, age range between 18 and 40 years, ability to read and write, Receiving IVF/ICSI for the first or second times.

### Exclusion criteria

Volunteers referred for receiving donor eggs, under treatment for other chronic diseases, sudden surgery or emergency statues, stress and incidents that disrupt the individual’s psychological and nervous status.

## Data collection instruments

The participant’s information will be collected using approved standard questionnaires. The Health Promoting Lifestyle Profile-II (HPLP-II) is the questionnaire that employed to determine the life status before and after the intervention. It was developed by Walker [32] and its validity and reliability are validated by Mohammadi et al., in Iran [33]. Stress and anxiety will be measured using Beck’s standard inventory [34], and it is validated by Mohammadkhani in Iran [35]. The IPAQ will be employed to measure the physical activity status [36, 37]. The frequencies with which food items are consumed will be assessed using the Food Frequency Questionnaire (FFQ) [37, 38]. Finally, the demographic information will be obtained by a researcher-made demographic questionnaire containing items related to age, weight, height, BMI, occupation, miscarriage, stillbirth, etc. The questionnaire will be completed at the beginning of the first visit. The subsequent assessment will be carried out within the specified time that will choose in expert panel after the intervention.

### Sampling, randomization, and blinding methods

After selecting the initial samples, the permuted block randomization will be done using a randomized block design with four blocks by a statistics consultant from a valid website (Sealed Envelope.Com or Randomization.Com). Then, the participants will be divided into two groups per the 1:1 allocation ratio. In this way, after listing all possible situations, 75 blocks of 4 will be created based on the sample size of 300 people. Then, by assigning a number to each of them, using a computerized random number table, with a ratio of 1:1, people will be divided into two intervention and control groups. Then, the subjects will be divided into two intervention and control groups. The numbers are placed in sealed envelopes in the order of each block generated. Each eligible patient who enters the room is then given an envelope. After that, they are assigned to one of these groups. Patient information will be recorded by the research team and confidentiality will be respected.

## Data analysis

The data obtained from the first stage will be analyzed using a meta-analysis tool in RevMan. The data of the clinical trial will be assessed using descriptive and analytical statistics by SPSS software. According to the type of variables, chi-square tests for descriptive variables, determining the average statistical difference of two groups by t-test for independent groups will be used. Regression tests will be performed for the relationship between the variables and the normality of the data will be determined first Findings of the clinical trial will be included in a scientific article following academic standards or will become available in scientific meetings.

## Discussion

Health-promoting lifestyle training program comprises a set of consistent activities, such as recommendations and training of skills regarding physical activity, diet, stress and anxiety adjustment methods, sleep hygiene, sleep disorders, adjusting meal portions, improving interpersonal relationships, considering self-actualization, paying attention to psychological health, learning methods of dealing with life problems, and avoiding general health risk factors. A couple is defined as a man and a woman in an emotional and sexual relationship, and there is an economic interaction between them. Couples’ lives and their interactions with each other affect their fertility health. One of the advantages of this study is the inclusion of infertile couples instead of individuals, which can improve the process of promoting health. In addition, they can reduce the pressure caused by infertility issues with collaboration. Another advantage of this study is its cost-effectiveness. Furthermore, couples could support each other and resolve problem together. The researcher will be in contact with the patients through face-to-face meeting in a private room in the infertility clinic or in other ways such as text messages, phone calls and social networking groups, providing daily relevant content and according to their needs and questions to is constantly in touch. This could be another benefit of this study. Furthermore, this study will be conducted by reviewing texts and methods examined up to the present and selecting the method with the highest score. One of the limitations of the study is the limited time for infertile people. Although scattered lifestyle training programs are considered separately for infertile women in Iran, the combined lifestyle training program is rarely used in infertility clinics. We hope that the results of this study will lead to the utilization and improvement of the health of infertile couples.

## Data Availability

Study data will be made available at completion of the study.

## Abbreviations

ART: Assisted reproductive technique
IVF: In vitro fertilization
ICSI: Intra cytoplasmic sperm injection
HPLII: Health promoting lifestyle II
BDI: Beck depression inventory
FFQ: Food frequency questionnaire
IPAQ: International physical activity questionnaire
NCD_S_: Non communicable diseases
FBDG: Food based dietary guideline
SPSS: Statistical Package for the Social Sciences
DASH: Dietary approach to stop hypertension
ESHRE: European Society of Human Reproduction and Embryology
SPIRIT: Standard Protocol Items: Recommendations for Interventional Trials
